# When HSFs bring the heat—mapping the transcriptional circuitries of HSF-type regulators in *Candida albicans*

**DOI:** 10.1128/msphere.00644-23

**Published:** 2024-12-20

**Authors:** Sadri Znaidi

**Affiliations:** 1 Institut Pasteur de Tunis, University of Tunis El Manar, Laboratoire de Microbiologie Moléculaire, Vaccinologie et Développement Biotechnologique, Tunis, Tunisia; 2 Institut Pasteur, INRA, Département Mycologie, Unité Biologie et Pathogénicité Fongiques, Paris, France; University of Georgia, Athens, Georgia, USA

**Keywords:** transcription factors, *Candida albicans*, *Saccharomyces cerevisiae*, functional genomics, systems biology, ChIP-seq, RNA-seq, bioinformatics, yeast genetics, filamentation, transcriptional regulation, heat-shock factors

## Abstract

Heat shock factor (HSF)-type regulators are stress-responsive transcription factors widely distributed among eukaryotes, including fungi. They carry a four-stranded winged helix-turn-helix DNA-binding domain considered as the signature domain for HSFs. The genome of the opportunistic yeast *Candida albicans* encodes four HSF members, namely, Sfl1, Sfl2, Skn7, and the essential regulator, Hsf1. *C. albicans* HSFs do not only respond to heat shock and/or temperature variation but also to CO_2_ levels, oxidative stress, and quorum sensing, acting this way as central decision makers. In this minireview, I follow on the heels of my mSphere of Influence commentary (2020) to provide an overview of the repertoire of HSF regulators in *Saccharomyces cerevisiae* and *C. albicans* and describe how their genetic perturbation in *C. albicans*, coupled with genome-wide expression and location analyses, allow to map their transcriptional circuitry. I highlight how they can regulate, in common, a crucial developmental program: filamentous growth.

## INTRODUCTION

All living organisms quickly and efficiently respond to environmental stress. The prompt induction of a transcriptional program that protects living cells from major insults, including those affecting their critical constituents, such as macromolecules and their building blocks, is tightly regulated by key stress-responsive transcription factors (TFs). For example, in the eukaryotic model organism *Saccharomyces cerevisiae*, the response to general stress is orchestrated by two closely related TFs, named Msn2 and Msn4 (multicopy suppressor of *SNF1* mutation proteins 2 and 4, respectively) (1). Both Msn2 and Msn4 carry a C_2_H_2_ zinc finger DNA-binding domain that binds DNA at stress-response elements and G4 quadruplexes located in the promoters of genes responding to a broad variety of stress signals (1, 2). They control the expression of most of the genes that are upregulated upon heat stress, osmotic stress, and carbon starvation stress (3) through an elegant mechanism whereby both regulators undergo nuclear-cytoplasmic shuttling dynamics (i.e., dynamic pulses) (4, 5). In addition to Msn2 and Msn4, Hsf1 (heat shock factor 1) plays a central role in cellular heat shock response (HSR), an evolutionarily conserved pathway that has been identified in all kingdoms of life, ranging from prokaryotes to humans (6). In *S. cerevisiae*, Hsf1 is essential for cell survival (7), and its target promoters extensively overlap with those of Msn2 and Msn4 (8). Indeed, the combined conditional nuclear depletion of Hsf1 and deletion of *MSN2* and *MSN4* make yeast cells unable to initiate the core stress-responsive program (9).

*C. albicans* is an opportunistic yeast colonizing the oral cavity, the gastrointestinal tract, and the vaginal mucosa in healthy individuals (10–12). In a disease context or in persons with a severely compromised immunity, it can express pathogenicity traits, translocate into the bloodstream (i.e., candidemia), and reach internal organs to cause life-threatening invasive infections (13). The ability of *C. albicans* to thrive within different niches of the human body is attributable to many TFs that control key fitness attributes, as well as developmental processes, such as white-opaque switching, chlamydosporulation, filamentation, and biofilm formation (14–19). As it alternates between commensal and pathogenic lifestyles, *C. albicans* “learned” to adapt to humans since its divergence with *C. dubliniensis* from their common ancestor thought to have occurred ~20 million years ago (20–22). But, unlike *S. cerevisiae*, *Schizosaccharomyces pombe*, or *Candida glabrata* (3, 23, 24), *C. albicans* is devoid of a large core stress response (25). Yet, the *C. albicans* genome does encode structural homologs of the Msn2 and Msn4 regulators of the core stress response pathway in *S. cerevisiae*, named Msn4 (CaMsn4) and Mnl1 (for Msn2- and Msn4-like). While Msn2 is syntenically homologous to CaMsn4, *S. cerevisiae* Msn4 has no syntenic homolog in *C. albicans*. Mnl1 is rather syntenically homologous to *S. cerevisiae* Com2 (Cousin of Msn2, YER130C), an orphan Msn2-like gene regulating the response to sulfur dioxide (26). Anyhow, CaMsn4 and Mnl1 do not regulate major stress response pathways in *C. albicans* (27, 28), pointing to transcriptional rewiring as a potential reassigner of their functions. Still, *C. albicans* copes with stress while thriving within the human body (29) and expresses a bona fide HSR that is regulated by the evolutionarily conserved heat shock transcription factor Hsf1 (30).

## HSF1 AND HSF-TYPE REGULATORS IN *SACCHAROMYCES CEREVISIAE*

Hsf1 regulatory function is considered as a prototypal model for explaining how eukaryotes quickly respond to stress (31). In yeast, Hsf1 is a promoter-resident protein that functions as a trimeric TF under both basal and stress-inducing conditions (32). It binds to a conserved sequence, nGAAn, located in distinct iterative repeat motifs, called heat shock elements (HSEs), on the promoter of target genes; many of which encode heat shock proteins (HSPs) (33). HSPs are a specific set of stress-protective proteins that counteract proteotoxic insults and help to maintain proteostasis (i.e., protein homeostasis) by preventing or even reversing the aggregation of unfolded proteins (34). Hsf1 regulates both basal and stress-induced expression of HSPs, with its basal activity being required to drive high levels of chaperone expression needed to sustain growth in yeast (35). Yet, out of hundreds of genes upregulated upon temperature increase, only a fraction (less than 50 genes) is directly regulated by Hsf1, suggesting that it controls a compact transcriptional program to maintain cellular proteostasis (35). In basal condition, Hsf1 directly interacts with its repressor, the chaperone Hsp70, which participates in a negative feedback loop that ensures appropriate coordination of the HSR with environmental conditions (36, 37). Following Hsf1 activation, Hsp70 is titrated away from its repressive interaction with Hsf1, leading to a strong upregulation of HSPs, including *HSP70* itself (36, 37). Once homeostasis is restored, Hsp70 binds to Hsf1 and inactivates the HSR, completing this way the negative feedback loop (36, 37).

**Fig 1:**
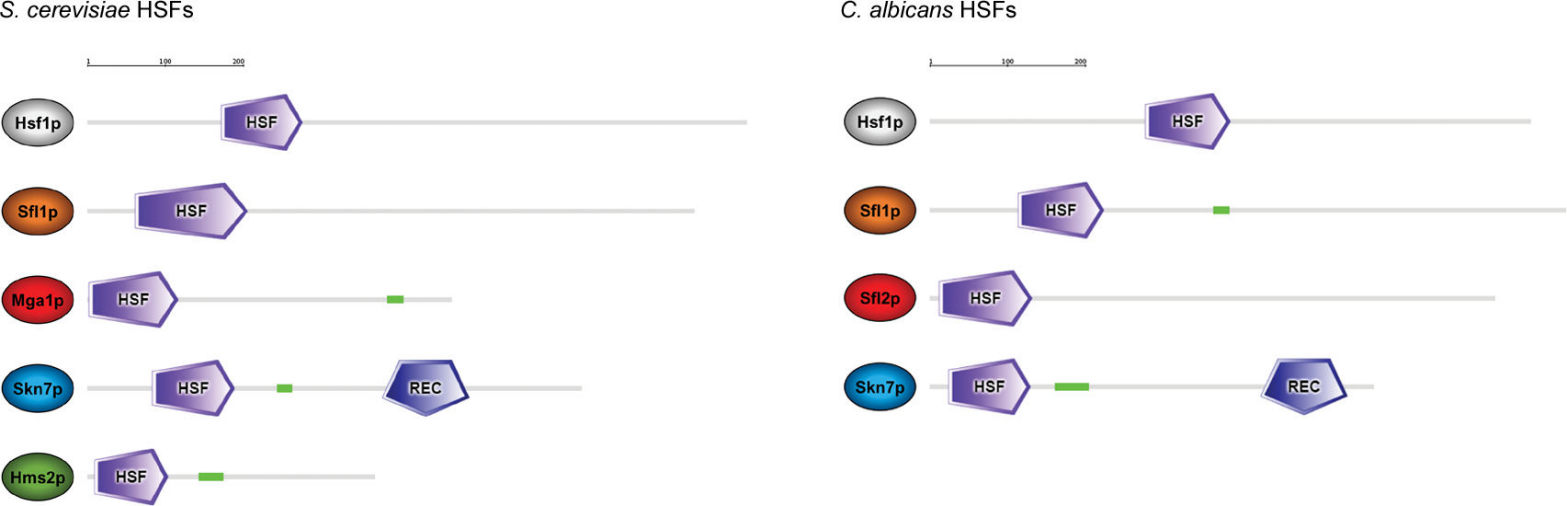
Repertoire of HSF-type regulators in *Saccharomyces cerevisiae* and *Candida albicans*. The *S. cerevisiae* (left panel) and *C. albicans* (right panel) genomes encode 5 and 4 proteins, respectively, with various lengths (light gray lines with sizes proportional to the size of the proteins, the scale bar on top of each panel representing 200 amino acids) carrying an HSF-type domain (HSF, purple shape), named Hsf1p (light gray oval), Sfl1p (orange oval), Mga1p/Sfl2p (red oval), Skn7p (blue oval), and Hms2p (absent in *C. albicans*, green oval). The Skn7p proteins also carry a receiver domain of the two-component phosphorelay signaling pathway (REC, blue shape) at their C-terminus, which is important for transcriptional regulation. A coiled-coil region is predicted in a subset of HSF regulators (green gaps). Protein depictions and domain annotations are taken from the Simple Modular Architecture Research Tool web resource (https://smart.embl.de) (62).

A hallmark of Hsf1 is that it carries a DNA-binding domain consisting of a four-stranded winged helix-turn-helix domain (38) considered as the signature domain for heat shock factors (HSFs) (39–41). The HSF domain is annotated as “HSF_DNA-bind” and “HSF” at the protein families (Pfam [42], now hosted by InterPro database) and the Simple Modular Architecture Research Tool (SMART [43]) databases, with accession numbers IPR000232 and SM00415, respectively. A search of the *S. cerevisiae* proteome allows to retrieve five hits carrying the HSF signature (Fig. 1, left panel), namely, Hsf1, Sfl1, Mga1, Skn7, and Hms2. Skn7 is a two-component response regulator that participates in the cell wall and oxidative stress response pathways in *S. cerevisiae* (44–48). Skn7 is also required for the induction of heat shock genes by oxidative stress, and it was shown to bind to HSEs and to physically interact with Hsf1 (49). Sfl1 is another HSF-type regulator capable of binding to HSEs, *in vitro* (50), and positively regulating the expression of a subset of HSPs, including *HSP26*, *HSP30*, *HSP104*, and *SSA4* (50–52). Yet, Sfl1 main function in *S. cerevisiae* is to act as a transcriptional repressor of flocculation (52–55), pseudohyphal/invasive growth (56–58), and biofilm formation (i.e., fluffy colony formation) (59). The two remaining HSF-type regulators, namely, Mga1 and Hms2, together with Skn7, were shown to enhance pseudohyphal growth when overexpressed (60). Hms2 is a structural paralog of Skn7 that presumably arose from the whole-genome duplication event, but, unlike Skn7, it does not carry a response regulator/receiver domain (Fig. 1, left panel), and its function has not been fully characterized so far. On the contrary, Mga1 antagonizes the function of Sfl1 by positively regulating pseudohyphal growth and is part of an intricate transcriptional circuitry that includes master regulators of filamentous growth Ste12, Tec1, Sok2, Phd1, and Flo8, where it acts as a transcriptional network hub (61).

Based on this body of literature, it is likely that the HSF paralogs have diversified in yeast to regulate developmental processes, such as pseudohyphal/invasive growth, while retaining some of the functions exerted by Hsf1, including the transcriptional control of HSPs. Many fungi exhibit filamentous, pseudohyphal, or invasive growth as a means of foraging for nutrients. In the opportunistic yeast *C. albicans*, this developmental process is a hallmark of its ability to invade the host tissues and cause disease in humans, a process facilitated by the hypha-associated peptide toxin candidalysin (63–65) encoded by *ECE1*, which is one member of the so-called “hyphal-specific genes.”

The combined use of functional genomics technologies to map transcriptional regulatory networks and discover pathways critical for *C. albicans*’ adaptation to two disparate niches of the human body, the gastrointestinal tract (i.e., as a commensal colonizer) and the bloodstream (i.e., as a pathogen during systemic infection), was the subject of an mSphere of Influence commentary I wrote in 2020 (66). I described seminal work that influenced the direction of my research by the Noble group on two iron-responsive TFs, named Sfu1 and Sef1, whose antagonistic functions in regulating iron uptake reflect on *C. albicans*’ commensal versus pathogenic lifestyles (67). In this minireview, I follow on the heels of my mSphere of Influence commentary to describe how genetic perturbation of HSFs coupled with genome-wide location and expression analyses allow to map their transcriptional network. I further highlight how HSFs regulate, in common, a crucial pathogenicity trait in *C. albicans*: filamentous growth. I also show that the HSF network carries a core of two paralogous but antagonistic HSF regulators, Sfl1 and Sfl2, controlling the yeast-to-hyphae transition, together with two decision makers of stress response: Skn7 (response to oxidative stress) and Hsf1 (response to heat shock).

## REPERTOIRE OF HSF-TYPE REGULATORS IN *C*. *ALBICANS* AND THEIR FUNCTIONS

The *C. albicans* genome encodes four HSF paralogs, namely, Hsf1 (CaHsf1), Sfl1 (CaSfl1), Sfl2, and Skn7 (CaSkn7) (Fig. 1, right panel), that are syntenically homologous to *S. cerevisiae* Hsf1 (ScHsf1), Sfl1 (ScSfl1), Mga1, and Skn7 (ScSkn7), respectively (Fig. 1, *Candida* Gene Order browser database, http://cgob.ucd.ie/ [68]). Hms2, the Skn7 paralog in yeast has no homolog in *C. albicans* and, as mentioned above, it likely arose through a gene duplication event that occurred in an ancestor of *S. cerevisiae* after it diverged from *C. albicans*.

Unlike *S. cerevisiae*, which is ubiquitously found in the environment and is, therefore, facing sudden thermal transitions in response to which it acutely activates the HSR pathway, *C. albicans* colonizes thermally buffered niches within the human body. This means that CaHsf1 could rather act as a molecular thermostat responding to milder thermal transitions (30). Yet, CaHsf1 is also essential in *C. albicans* and it has indeed been shown to strongly upregulate the expression of HSPs through the canonical HSE motif ([nGAAn]_3_) in response to experimental heat shock and to moderately respond to cell wall stress but not to oxidative, osmotic, weak acid and pH stresses (69). Like ScHsf1, CaHsf1 maintains the basal expression of a subset of HSPs necessary for viability in the absence of heat shock (30, 69). Still, CaHsf1 is activated during thermal transitions that mimic fever (70), and its activation is important for *C. albicans* to express full virulence, *in vivo* (71). In *S. cerevisiae* and the ancestrally related yeast *Lachancea kluyveri*, Hsf1 physically associates with Hsp70 but not with the Hsp90 chaperone (36), contrasting with CaHsf1, which interacts with both Hsp70 and Hsp90 (72). CaHsf1 binds to the promoter of 49 genes in basal condition and to that of 55 additional targets upon heat shock (73), a behavior consistent with the “condition expanded” model of transcriptional networks, whereby regulators bind to a core set of target promoters under one condition but bind an expanded set of promoters under another condition (74). This mode of transcriptional regulation might reflect increased levels of the regulator available for DNA binding (74). Indeed, Ca*HSF1* undergoes positive transcriptional autoregulation (73). In addition to HSP-encoding genes and genes involved in protein folding, CaHsf1 directly controls the expression of oxidative stress-responsive genes, such as *CCP1*, *GPX2*, *GPX3*, *TRX1*, *TSA1*, and *TSA1B* (73). Strikingly, the overexpression of Ca*HSF1* activates filamentous growth in the absence of external cues (75). Combination of genome-wide expression and location analyses identified 227 and 170 directly upregulated and downregulated targets, respectively (75), indicating that CaHsf1 acts as both activator and repressor of gene expression. It is speculated that Ca*HSF1* overexpression induces a dose-dependent expansion of CaHsf1 direct targets that drives upregulation of positive regulators of filamentation, such as *BRG1* and *UME6* (75). It still remains possible that CaHsf1 responds to yet unknown signals to drive filamentous growth in *C. albicans*.

CaSfl1 is the functional homolog of ScSfl1 (76, 77). As stated above, ScSfl1 represses flocculation, pseudohyphal/invasive growth, and biofilm formation in baker yeast (52–59). An analogous function appears to have been retained in CaSfl1. The heterologous expression of Ca*SFL1* suppresses both the flocculent phenotype of a haploid *S. cerevisiae sfl1*Δ mutant and the pseudohyphal growth of a diploid *sfl1*Δ mutant (77). Consistent with a repressor function in *C. albicans* morphogenesis, deletion of Ca*SFL1* activates flocculation and hyphal development, correlating with the upregulation of hyphal-specific genes *ALS3*, *HWP1*, and *ECE1* (76, 77), whereas its overexpression represses hyphal growth (77). The consequences of the control of *C. albicans* filamentation by CaSfl1 extend beyond morphogenesis *per se*, as CaSfl1 was shown to be critical for repression of hyphal growth by quorum sensing molecules dodecanol (78), *Burkholderia* diffusible signal factor (78, 79), and mutanocyclin (80) during interspecies microbial interactions. It also represses pathogenic microcolony formation under flow (81) but appears to promote biofilm formation under acidic growth conditions (82, 83). CaSfl1 can act as both activator and repressor of gene expression. This has been suggested through genome-wide expression and location analyses of CaSfl1 (84), enabling mapping of the direct CaSfl1 regulon. CaSfl1 is a constitutively nuclear protein (76, 77) consistent with its role as a TF. Yet, one-hybrid assay did not detect any activator or repressor activity (76), indicating that DNA binding *per se* is not sufficient to dictate its function as an activator/repressor.

CaSfl1 has a structural paralog in *C. albicans*, named Sfl2. Intriguingly, although Sfl2 is syntenically homologous to Mga1, *SFL2* is able to functionally complement the *sfl1*Δ mutation but not the *mga1*Δ mutation in *S. cerevisiae* (85). Sfl2 is functionally antagonistic to CaSfl1, acting as a positive regulator of *C. albicans* filamentous growth (85, 86). *SFL2* was identified as one of the highly upregulated genes correlating with the rapid induction of filamentation on reconstituted human oral epithelium infected by *C. albicans* (86). *SFL2* deletion impairs hyphal growth under various filamentation-inducing conditions, whereas its overexpression is sufficient to activate filamentation in the absence of external cues (85, 86). Importantly, elevated temperature (37°C) and CO_2_ (5% CO_2_) have major impact on Sfl2 function (85, 87). Unlike CaSfl1, which is constantly expressed and localizes to the nucleus under various growth conditions (76, 77), Sfl2 expression and cellular localization are undetectable in rich medium at 25 or 30°C (85). At 37°C or under 5% CO_2_, *SFL2* expression is induced (85, 87), and Sfl2 rapidly accumulates in the nucleus (85). Therefore, despite their structural similarity, CaSfl1 and Sfl2 exert antagonistic functions. Their divergent role is also reflected in their different expression patterns, one being constitutively expressed and localized in the nucleus, and the other being induced by temperature/CO_2_ increase and/or other filament-inducing cues upon which it localizes to the nucleus (76, 77, 85, 86). Although one would expect the non-conserved portions between both regulators to carry the information for their divergent activity, it is their HSF DNA-binding domain that mediates their functional divergence (85). Indeed, swapping of the two HSF DNA-binding domains of CaSfl1 and Sfl2 is sufficient to almost completely reverse their activities (85). This finding hypothesizes that CaSfl1 and Sfl2 recognize divergent binding sites on the *C. albicans* genome. Mapping of the CaSfl1 and Sfl2 direct regulon reinforced such hypothesis (84). While Sfl2 binds to a DNA motif resembling that of *S. cerevisiae* Mga1, 5′-AnAATAGAA-3′ (where n indicates any nucleotide) (84), CaSfl1 appears to rather recognize an HSE motif similar to that of ScSfl1 and ScHsf1, 5′-TTCnnGAA-3′, or a more divergent motif, 5′-TCGAACCC-3′ (84). Yet, CaSfl1 and Sfl2 bind to the same set of target promoters, with binding enrichment peaks overlapping with each other (84). Sfl2 additionally binds to the promoter of hyphal-specific genes to activate their expression (84). It also binds to the promoter of a subset of the tricarboxylic acid cycle genes upon activation by 5% CO_2_, linking CO_2_ sensing by Sfl2 to hyphal growth (87). This reflects a transcriptional circuitry where CaSfl1 and Sfl2 act as central “switch on/off” proteins to control filamentous growth in *C. albicans*.

CaSkn7 is the syntenic homolog of ScSkn7 encoded by the suppressor of *kre* null 7 (*SKN7*) gene and initially isolated as a multicopy suppressor of a *kre9* mutation linked to a defective cell wall biosynthesis (45). Sc*SKN7* was also isolated as *POS9* in a screen for mutants resulting in sensitivity to hydrogen peroxide (88). The Skn7 proteins are highly conserved among Saccharomycetes (89). They carry a receiver domain of the “two-component” His→Asp phosphorelay signaling pathway, where component one is a histidine kinase, and component two is the response regulator—in this case Skn7—with an intermediate histidine phosphotransfer protein, named Ypd1, in *S. cerevisiae* (44, 45, 89–91). The receiver domain is important for the Skn7 transcriptional activity, a type of regulation distinguishing it from other eukaryotic TFs (90). As stated above, ScSkn7 regulates cell wall integrity and the oxidative stress response (48, 92). However, these two functions are mechanistically uncoupled. The one regulating cell wall integrity requires the Sln1-mediated two-component phosphorelay pathway, whereas the other one is independent of two-component signaling (46, 93). In fact, ScSkn7 cooperates with a key regulator of the oxidative stress response, Yap1, requiring ScSkn7 receiver domain for physical interaction (94–97). It is thought that this may help partition ScSkn7 to oxidative stress response promoters when the Yap1 protein accumulates in the nucleus (97). ScSkn7 also cooperates with ScHsf1 in the maximal induction of the expression of HSP genes during oxidative stress (49), reflecting a complex transcriptional regulatory network of ScSkn7. Like ScSkn7 in *S. cerevisiae* (60), CaSkn7 overexpression activates filamentous growth in *C. albicans* in the absence of external cues (98, 99). CaSkn7 is required for hyphal morphogenesis on several filamentation-inducing solid media (100) but not in liquid medium (98), suggesting a specific role in surface-induced morphogenesis. It also protects *C. albicans* against oxidative stress mediated by exposure to H_2_O_2_ or *tert*-butyl hydroperoxide (98, 100); however, it is not phenotypically required for adaptation to cell wall and osmotic stresses (98). Unlike ScSkn7, CaSkn7 does not appear to rely on the *C. albicans* Yap1 homolog, Cap1, to efficiently respond to extracellular oxidative stress (98). Still, this function appears to be independent of the CaSln1 two-component pathway, like in *S. cerevisiae* (98). Interestingly, CaSkn7 also protects *C. albicans* from intracellular oxidative stress generated by the intracellular accumulation of reactive oxygen species during filamentous growth on solid medium (98). Intriguingly, this specific function appears to depend on the CaSln1 two-component signaling pathway (98). Hence, some functional Skn7 circuits could have been rewired between *S. cerevisiae* and *C. albicans* probably as a consequence of their divergent lifestyles.

## MAPPING THE TRANSCRIPTIONAL NETWORK OF HSF-TYPE REGULATORS IN *C. ALBICANS*

**Fig 2:**
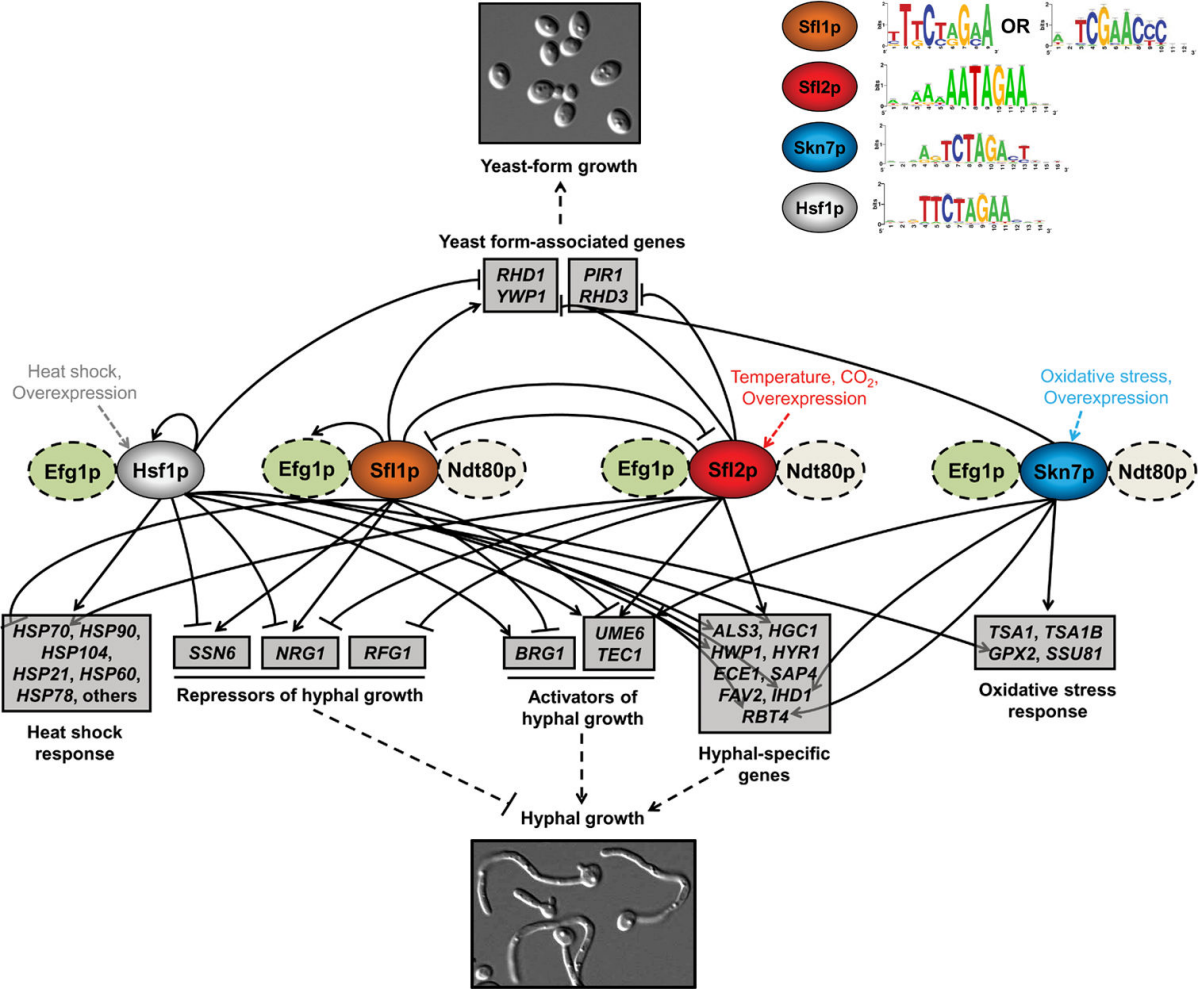
*Candida albicans* HSF network. Hsf1p (light gray oval), Sfl1p (orange oval), Sfl2p (red oval), and Skn7p (blue oval) directly regulate, in common, the transcription (arrowed lines, direct upregulation; blunt lines, direct downregulation) of many target genes (gray boxes) grouped into six categories: heat shock response genes, repressors of hyphal growth, activators of hyphal growth, hyphal-specific genes, oxidative-stress response genes, and yeast form-associated genes. They do so by binding to their respective DNA-binding motifs illustrated on the top right part of the figure. Sfl1p and Sfl2p exert a direct negative regulation on the expression of each other, while Hsf1p undergoes positive transcriptional autoregulation. The execution of these transcriptional regulatory interactions allows to control the commitment (dashed lines; blunt, inhibition; arrowed, activation) of *C. albicans* to form hyphae (bottom microscopy image) or maintain the yeast form growth (top microscopy image). Ndt80p and/or Efg1p (light green and white ovals, respectively; dashed lines indicate hypothetical physical and/or functional interaction) co-operate with HSF regulators to transcriptionally modulate the expression of target genes. Binding and gene expression data were taken from references 75 (Hsf1), 84 (Sfl1, Sfl2), and 98 (Skn7).

Based on the accumulated lines of evidence presented above, it is clear that each of the four *C. albicans* HSF regulators has specific functions in the cell while regulating, in common, *C. albicans* morphogenesis. CaHsf1 regulates the heat-shock stress response (69–72, 75), while CaSkn7 regulates both extracellular and intracellular oxidative stress responses (98, 100). On the contrary, CaSfl1 and Sfl2 antagonistically control microcolony formation (81) and filamentous growth in response to temperature (84–86). The overexpression of the three HSF regulators CaHsf1, CaSkn7, and Sfl2 activates filamentous growth, while CaSfl1 overexpression inhibits it (75, 84, 98). One would expect that the four TFs share common direct targets acting as effectors or key regulators of filamentous growth. The data gathered from genome-wide expression and location analyses can be extensively mined in order to map, integrate, and conceptualize the direct regulatory interactions occurring between these TFs and their target genes (66, 101), making better sense of transcriptional networks (102). In three key studies, the direct regulon of the four HSFs CaSfl1, Sfl2, CaHsf1, and CaSkn7 has been characterized by genome-wide expression and location analyses following a similar genetic perturbation approach (i.e., by gene overexpression) (75, 84, 98). By integrating the resulting data, I mapped a simplified transcriptional circuitry of the four HSF regulators, highlighting how they can directly regulate, in common, filamentous growth in *C. albicans* (Fig. 2). CaSfl1 and Sfl2 are central in the resulting network (Fig. 2), acting as molecular switch on/off proteins of the yeast–hyphal transition (84). The CaSfl1/Sfl2 network has been mapped and described in detail by Znaidi et al. (84), allowing it to serve as a backbone for the resulting network (Fig. 2). The CaSkn7 direct regulon overlaps to some extent with that of the CaSfl1/Sfl2 regulon (Fig. 2) by co-binding to the promoter of a subset of CaSfl1/Sfl2 targets in order to activate filamentous growth (*UME6*, *TEC1*, *IHD1*, and *RBT4*) and repressing yeast-form growth (*RHD1* and *YWP1*) while protecting the cells against intra/extracellular oxidative stress through direct upregulation of *TSA1*, *TSA1B*, *GPX2*, and *SSU81* (98) (Fig. 2). CaHsf1 comes as a fourth member of this network by activating hyphal growth through the direct upregulation of positive regulators of filamentation (*BRG1*, *UME6*, and *TEC1*) and a subset of hyphal-specific genes (*ALS3*, *HGC1*, *HWP1*, *IHD1*, and *RBT4*) while directly repressing yeast form-associated genes (*RHD1* and *YWP1*) and genes encoding repressors of morphogenesis (*SSN6* and *NRG1*) (Fig. 2). CaHsf1 also directly controls the expression of heat shock-responsive genes (*HSP70*, *HSP90*, *HSP104*, and others), as well as a subset of CaSkn7 direct targets involved in oxidative stress response (*GPX2*), contributing this way to its primary function in response to stress (Fig. 2).

As stated above, the binding sites of the four HSF regulators are expected to be divergent (Fig. 2). CaSfl1 binds to a motif similar to ScSfl1/ScHsf1 HSE, 5′-TTCnnGAA-3′ (where n designates any nucleotide) or to the more divergent motif, 5′-TCGAACCC-3′ (84) (Fig. 2), whereas CaSfl2 recognizes a motif reminiscent of that of *S. cerevisiae* Mga1, 5′-AnAATAGAA-3′ (84) (Fig. 2). Intriguingly, CaSkn7 appears to rather bind to a shorter version of the canonical HSE motif, 5′-TCTAGA-3′ (98) (Fig. 2), similar to that of *S. cerevisiae* Abf2 involved in the regulation of respiratory growth, osmotic stress, and mitochondrial DNA maintenance and packaging (103, 104). On the contrary, CaHsf1 binds to a clear HSE canonical motif consisting of 5′-TTCTAGAA-3′ (Fig. 2) obtained by running the deduced peak sequences encompassing 500 base pairs centered on CaHsf1 peak locations obtained by Veri et al. (75) on the Regulatory Sequence Analysis Tool peak motif algorithm (105). Strikingly, I noticed the co-enrichment of the Efg1 motif in my analyses (data not shown), a finding correlated with the previous observation of *EFG1* requirement for *C. albicans* morphogenesis following Ca*HSF1* genetic perturbation (75). Interestingly enough, the enrichment of CaSfl1, Sfl2, and CaSkn7 motifs in ChIP-chip/seq data also occurs with the co-enrichment of binding motifs for Efg1 (5′-TGCA-3′) and Ndt80 (5′-ACACAAAA-3′) (84, 98) (Fig. 2), suggesting the occurrence of physical/functional interactions between the HSF and key regulators of *C. albicans* morphogenesis. Indeed, co-immunoprecipitation experiments confirmed that CaSfl1/Sfl2 is physically associated with Efg1 (84). Whether CaSkn7 and CaHsf1 also physically interact with Efg1/Ndt80 remains to be determined (Fig. 2). These interactions could be essential to inducing hyphal growth. Indeed, genetic interaction analyses are supportive of such hypothesis since the overexpression of Ca*SKN7* or Ca*SFL2* requires a functional *EFG1* gene to induce filamentous growth (84, 98). Hence, all *C. albicans* HSF regulators are likely to form a transcriptional regulatory complex with additional regulators of *C. albicans* morphogenesis. One open question that remains to be answered pertains to the precise sequence of molecular events governing the HSF transcriptional activity. Another question is whether HSF regulators in *S. cerevisiae* could form a regulatory complex with additional morphogenesis regulators. While this is clearly the case for Mga1, which is part of an intertwined circuitry regulating filamentous growth with other morphogenesis regulators in *S. cerevisiae*, such as Flo8 (61), it is currently unknown whether ScSfl1 functionally interacts with Mga1. Likewise, it is unclear how ScSkn7 regulates pseudohyphal growth in *S. cerevisiae* and whether or not it cooperates with the remaining HSF regulators in this process.

## CONCLUDING REMARKS

The ChIP-seq and RNA-seq technologies are cornerstones for constructing transcriptional regulatory networks and mapping higher-order circuitries using powerful bioinformatics tools and integrative analyses (15, 66, 101, 106–110). The observations made above and the transcriptional regulatory network shown in Fig. 2 are based on genetic perturbation of *C. albicans* HSFs using a gene overexpression approach, a genetic strategy that allows to identify TF targets and DNA-binding motifs without prior knowledge of TF inducers and is successfully used to accurately predict the function of unknown TFs (109, 111). When combined with ChIP/RNA-seq and gene-set enrichment analyses for mapping overrepresented pathways, one could identify direct transcriptional signatures that provide further clues on TF function and potential inducers of TF activity (16, 66, 84, 98, 101, 112–115). Such strategy could be used in the future for comprehensively mapping transcriptional regulatory networks, inferring TF function, and testing it experimentally. In addition, the potential for mapping transcriptional circuitries goes beyond the conceptual modeling of the structure of TF-target gene networks (116) to include the accurate prediction of regulatory protein complexes through motif co-enrichment analyses (84, 98), followed by experimental validation by genetic and protein–protein interaction analyses. Current and future discoveries in this field reinforce the trending concept of “transcriptional droplets,” a new model of gene control whereby transcriptional condensates concentrate large amounts of TFs at key target genes, forming higher-order TF complexes (117–121). Transcriptional droplets refer to the phase separation of TFs containing intrinsically disordered regions (IDRs) characterized by their lack of stable secondary or tertiary structure (120). Interestingly, HSF regulators CaHsf1 and CaSfl2 possess putative IDRs in their deduced amino acid sequences predicted by the MobiDBLite algorithm at the *Candida* Genome Database. The presence of IDRs may indicate the potential for interaction with other regulators through the formation of condensates. Clearly, we are at the beginning of a fascinating new era in the field of transcriptional regulation.

## AFTER EPUB

*[This article was published on 20 December 2024 with an error the title. The title was corrected in the current version, posted on 3 January 2025.]*
